# Snail suppresses cellular senescence and promotes fibroblast‐led cancer cell invasion

**DOI:** 10.1002/2211-5463.12300

**Published:** 2017-09-11

**Authors:** Satoshi Furuya, Kaori Endo, Akiko Takahashi, Keiji Miyazawa, Masao Saitoh

**Affiliations:** ^1^ Department of Biochemistry Interdisciplinary Graduate School of Medicine University of Yamanashi Japan; ^2^ Research Training Program for Undergraduates Interdisciplinary Graduate School of Medicine University of Yamanashi Japan; ^3^ Division of Cancer Biology The Cancer Institute Japanese Foundation for Cancer Research Tokyo Japan; ^4^ Center for Medical Education and Sciences Interdisciplinary Graduate School of Medicine University of Yamanashi Japan

**Keywords:** cellular senescence, EMT, Snail

## Abstract

Snail, a zinc finger transcription factor, induces an epithelial–mesenchymal transition (EMT) in various cancer and epithelial cells. We investigated the function of Snail (*SNAI1*) by downregulating its expression with short interfering RNA (siRNA). Suppression of Snail expression induced cellular senescence in several cancer cells and in normal fibroblast IMR90 cells. Cancer progression is facilitated by fibroblasts, so‐called fibroblast‐led cancer cell invasion. Snail‐silenced cancer cells exhibited reduced motility, which was further decreased by cocultivation with Snail‐silenced IMR90 cells. Our data suggest that cell motility and cellular senescence, which are regulated by Snail in cancer cells and fibroblasts, modulate fibroblast‐led cancer cell invasion. Therefore, we propose that local suppression of Snail in cancer and the cancer microenvironment represents a potent therapeutic strategy.

**Abbreviations**
CAF: cancer‐associated fibroblast
EMT: epithelial–mesenchymal transition
qRT‐PCR: quantitative RT‐PCR
SASP: senescence‐associated secretory phenotype
SA‐βgal: senescence‐associated β‐galactosidase
siRNA: short interfering RNA
TGF‐β: transforming growth factor‐β

The epithelial–mesenchymal transition (EMT) is a developmental process involving loss of epithelial features and gain of mesenchymal phenotypes 1. Invasion by tumor cells is associated with loss of cell–cell interaction along with acquisition of migratory properties, and is often accompanied by the EMT 2. Transforming growth factor‐β (TGF‐β) acts as a key mediator of the EMT during physiological processes by regulating expression of several transcription factors, including the zinc finger factor Snail. Snail, which is encoded by *SNAI1*, is upregulated through the TGF‐β–Smad signaling pathway, which is dramatically activated in cooperation with signals from active Ras signals or growth factors 3. Snail plays crucial roles in processes of embryonic development, and mutant mouse embryos lacking this protein die at gastrulation due to a deficiency in the EMT 4, 5. In cancer cells, EMT induced by Snail confers invasiveness, drug/stress resistance, and immunosuppression 2, 6. In addition to cancer cells, fibroblasts and cancer‐associated fibroblasts also express Snail 7 and cooperatively promote cancer progression by remodeling cancer microenvironment and leading chains of invading cancer cells into adjacent tissues 8. Although Snail has been extensively studied by gain‐of‐function analysis, detailed loss‐of‐function studies have not been previously performed, but such analysis could provide valuable information for molecularly targeted therapy to prevent cancer progression.

During wound healing and in tissue inflammation, fibroblasts become activated and then revert back to a normal state or undergo apoptosis after the wound is healed 9. Activated fibroblasts at tumor sites are known as cancer‐associated fibroblasts (CAFs) 10. During cancer progression, fibroblasts and CAFs are the most abundant noncancerous cell types in the tumor stroma. Stromal fibroblasts and CAFs play crucial roles in cancer cell proliferation, survival, angiogenesis, and invasion. In 3D organotypic models, fibroblasts promote the collective invasion of cancer cells, which is known as fibroblast‐led (collective) cancer cell invasion 11. Fibroblasts and/or CAFs are always the leading cells in the invading cohort, with the collective cancer cells following behind. The GP130/JAK/STAT3 and RhoA/ROCK/MLC2 signaling pathways are constitutively activated in activated fibroblasts and CAFs and regulate fibroblast‐led cancer cell invasion via generation of fibrotic and tumorigenic cancer‐associated extracellular matrix 12. However, the detailed molecular mechanism underlying this process remains unclear.

Cellular senescence is defined as a permanent cell cycle arrest 13, 14. Senescent cells have several distinctive characteristics, including a ‘sunny‐side up’ shape, and expression of senescence‐associated β‐galactosidase (SA‐βgal) 15. Cellular senescence can be induced by stresses such as DNA‐damaging agents, and is also observed in nonimmortalized cells upon serial passage *in vitro*. Numerous biochemical and genetic studies have revealed that several different signaling pathways can induce cellular senescence, but these pathways eventually converge on activation of the retinoblastoma tumor suppressor gene product (RB) and its family members (p107 and p130). Once the RB family proteins are fully activated, particularly by the p16INK4A cyclin‐dependent kinase (CDK) inhibitor, the cell cycle arrest becomes irreversible and can no longer be revoked by subsequent inactivation of the RB pathway. These findings place the RB pathway at the heart of the cellular machinery responsible for the irreversibility of senescent cell cycle arrest 16, 17. Due to the frequent somatic mutation of *p16INK4A* in cancer, cellular senescence is often impaired in cancer cells, enabling malignant transformation. Similar to cellular senescence, cell death by apoptosis and necrosis also prevent cells from malignant transformation and uncontrollable proliferation. By contrast, however, senescent cells can survive and exert effects on the surrounding tissue via secretion of multiple factors, the so‐called senescence‐associated secretory phenotype (SASP) 18, 19. The SASP performs several functions, including sensitizing surrounding cells to senescence and promoting tissue development. Although the details and implications of this regulation have not been clarified, the SASP promotes tumorigenesis through the secretion of numerous bioactive molecules, including proinflammatory cytokines, chemokines, growth factors, and proteases, and can contribute to a procarcinogenic microenvironment. Therefore, depending upon the context, cellular senescence and the SASP contribute to multiple physiological functions, both beneficial and deleterious.

Snail overexpression attenuates the cell cycle and confers resistance to cell death induced by proapoptotic signals and withdrawal of survival factors in Madin–Darby canine kidney (MDCK) cells 20. Conversely, long‐term knockdown of Snail induces cellular senescence in prostate cancer cells 21. In this study, we found that Snail knockdown caused cellular senescence in several cancer cell lines and IMR90 normal fibroblasts. Consistent with previous observations on Ras‐induced cellular senescence, Snail siRNA controlled cellular senescence by regulating the AKT/p16INK4A/RB pathway. Conversely, overexpression of Snail and induction of Snail by TGF‐β inhibited cellular senescence. In addition, suppression of Snail expression reduced fibroblast‐led cancer cell invasion. Therefore, siRNA‐mediated suppression of Snail could serve as a therapeutic strategy in cancer cells.

## Materials and methods

### Cell culture, antibodies, and reagents

Panc‐1, MIAPaCa‐2, HEK293FT, Suit‐2, A549, and IMR90 cells were obtained from ATCC. Panc‐1, Suit‐2, A549, and MIAPaCa‐2 cells were cultured in Dulbecco's modified Eagle's medium (DMEM; Nacalai Tesque, Kyoto, Japan) in the presence of 10% FBS, 50 U·mL^−1^ penicillin, and 50 μg·mL^−1^ streptomycin (Nacalai Tesque). IMR90 cells were cultured in Eagle's minimum essential medium with Eagle's salts (EMEM; Wako Pure Chemical Industries, Osaka, Japan) supplemented with 10% FBS, 1 mm nonessential amino acids (Life Technologies, Carlsbad, CA, USA), and the same antibiotics. To produce lentivirus, HEK293FT cells were cultured in DMEM supplemented with 10% FBS, 50 U·mL^−1^ penicillin, 50 μg·mL^−1^ streptomycin, 2 mm L‐glutamine (Invitrogen, Carlsbad, CA, USA), 0.1 mm MEM nonessential amino acids (Invitrogen), and 1 mm MEM sodium pyruvate (Invitrogen). IMR90‐Ras cells were kindly provided by E. Hara and G. Peters. All cells were grown in a 5% CO_2_ atmosphere at 37 °C.

### Antibodies and reagents

Mouse monoclonal anti‐α‐tubulin antibody was purchased from Sigma‐Aldrich (St. Louis, MO, USA). Rat monoclonal anti‐Snail antibody, mouse monoclonal RB, and rabbit polyclonal anti‐phospho‐RB and phospho‐AKT antibodies were purchased from Cell Signaling Technology (Danvers, MA, USA). Rabbit anti‐p16INK4A antibody and rat anti‐HA (3F10) antibody were from Abcam (Cambridge, UK) and Roche (Indianapolis, IN, USA), respectively. Rabbit anti‐p21 antibody was from SantaCruz Biotechnology (Dallas, TX, USA). Transient transfection with siRNAs was performed using Lipofectamine RNAiMAX (Invitrogen). (Z)‐4‐Hydroxytamoxifen (4OHT) was obtained from Sigma‐Aldrich.

### RNA extraction, quantitative RT‐PCR analysis, and RNA interference

Total RNA was purified using the RNeasy Mini Kit (Qiagen, Valencia, CA, USA) and used for quantitative RT‐PCR (qRT‐PCR) analyses. Values were normalized against the corresponding levels of human hypoxanthine phosphoribosyltransferase 1 (HPRT1) mRNA. The primer sequences were described previously 22. The final concentration of the siRNAs was 10 nm. The sequences of the Snail siRNAs were as follows:

Snail#1: 5‐AGACCCACUCAGAUGUCAAGAAGUA‐3

Snail#2: 5‐CCUGUCAGAUGAGGACAGUGGGAAA‐3

### Lentiviral production and immunoblotting

The procedures used for lentiviral production, infection, and immunoblotting were previously described 23. Lentiviral infection was performed in cells seeded in a well of the tissue culture plate and repeated at least three times with lentiviruses, which were independently prepared for each experiment. Cells were lysed in lysis buffer solution [20 mm Tris/HCl, pH 7.5, 150 mm NaCl, 10 mm EDTA, 1 mm EGTA, 1% Nonidet P‐40, protease inhibitors (Nacalai Tesque)]. After measurement of protein concentrations with a BCA Protein Assay Kit (Pierce, Rockford, IL, USA), equal amounts of total protein per lane were subjected to SDS/PAGE, followed by semidry transfer of the proteins to Fluoro Trans W membrane (Pall, Glen Cove, NY, USA). Nonspecific binding of proteins to the membrane was blocked by incubation in TBS‐T buffer (50 mm Tris/HCl, pH 7.4, 150 mm NaCl, and 0.1% Tween‐20) containing 5% skim milk. Immunodetection was performed with the ECL blotting system and Luminescent Image Analyzer (LAS4000; Fujifilm, Tokyo, Japan).

### Senescence‐associated β‐galactosidase staining

Senescence‐associated β‐galactosidase (SA‐β‐gal) staining was performed as described previously 15. Briefly, cells were washed in PBS, fixed in 2% formaldehyde/0.2% glutaraldehyde, washed in PBS again, and stained with staining solution (1 mg·mL^−1^ X‐gal, 40 mm citric acid/sodium phosphate, 5 mm potassium ferrocyanide, 5 mm potassium ferricyanide, 150 mm NaCl, and 2 mm MgCl_2_) at 37 °C for 3–12 h. To evaluate senescence, SA‐β‐gal‐positive and SA‐β‐gal‐negative cells were photographed on a TS100 microscope (Nikon, Tokyo, Japan) at 100× magnification and counted in five random independent fields.

### Cell invasion and proliferation assays

Boyden chamber migration assays were conducted using transparent PET membrane 24‐well 8.0‐μm pore size cell culture inserts (BD Falcon, Franklin Lakes, NJ, USA) coated with collagen type I‐C (Nitta Gelatin, Osaka, Japan). After the cells were seeded in triplicate on the inserts, the cells that had not invaded the lower surfaces of the filters were removed from the upper faces of the filters using cotton swabs. Cells that had invaded into the lower surfaces of the filters were fixed in methanol and acetone and stained with Giemsa. Invasion was quantitated by visually counting the photographed cells, and evaluated by statistical analysis.

## Results

### Regulation of cellular senescence in cancer cells by Snail

**Figure 1:**
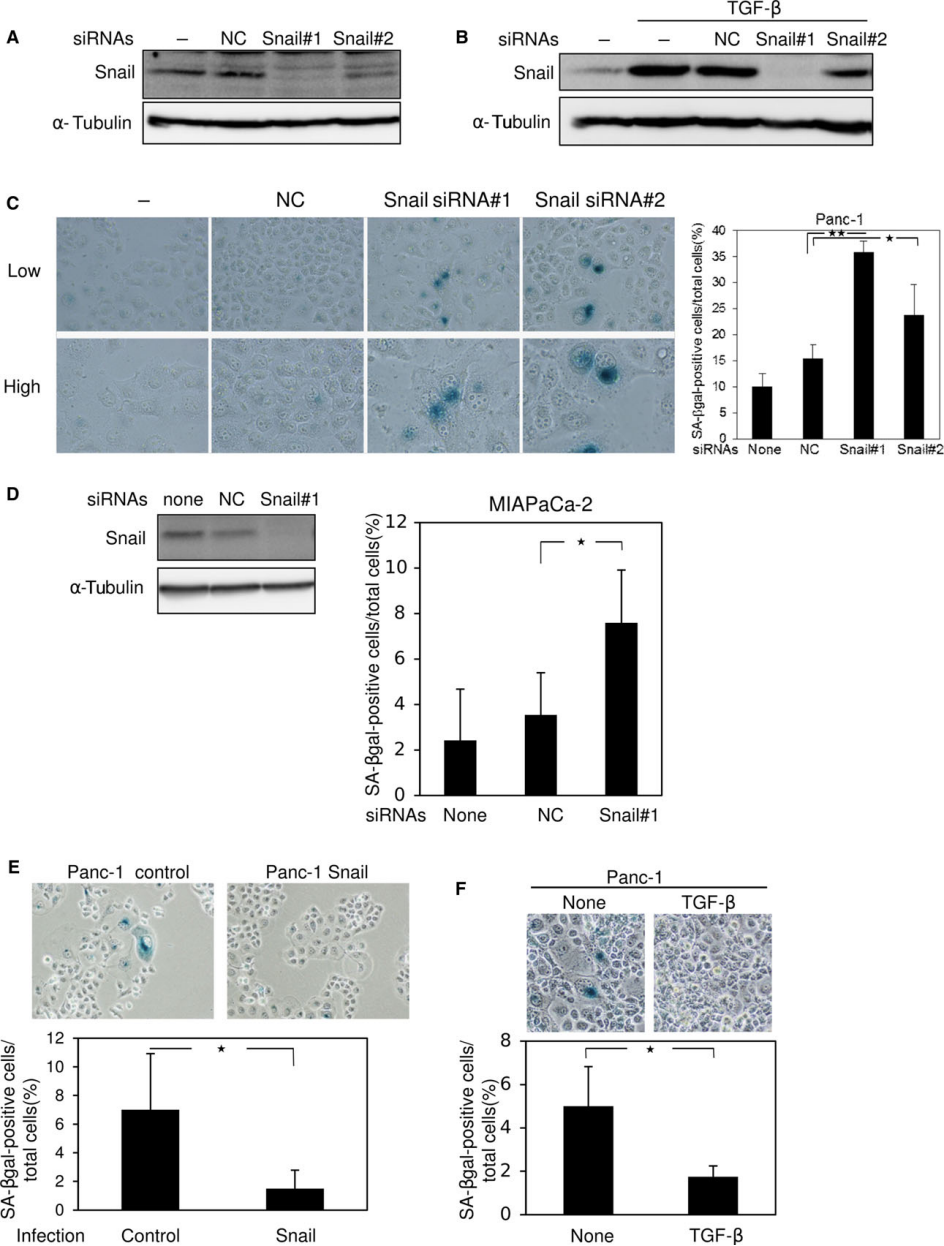
Cellular senescence by Snail knockdown in pancreatic cancer cells. (A and B) Forty‐eight hours after transfection of control siRNA (NC) or two kinds of siRNAs against human Snail (#1 and #2) into Panc‐1 cells, the level of Snail protein was examined by immunoblotting. Cells were treated with 1 ng·mL^−1^ of TGF‐β for 3 h (B). α‐Tubulin levels were monitored as a loading control. Results are representative of at least three experiments. (C) Three days after transfection with control siRNA (NC) or Snail siRNAs (#1 and #2), Panc‐1 cells were analyzed by SA‐β‐gal staining, and photographs were taken (left panel) to count the number of SA‐β‐gal‐positive cells (right panel). Low, low magnification (×100). High, high magnification (×200). *P < 0.05, **P < 0.01, ANOVA test. (D) Forty‐eight hours after transfection of control siRNA (NC) or Snail siRNA (#1) into MIAPaCa‐2 cells, the level of Snail protein was examined by immunoblotting. α‐Tubulin levels were monitored as a loading control (left panel). Three days after transfection with control siRNA (NC) or Snail siRNAs (#1), MIAPaCa‐2 cells were analyzed by SA‐β‐gal staining, and photographs were taken to count the number of SA‐β‐gal‐positive cells (right panel). *P < 0.05, ANOVA test. (E) Panc‐1 cells infected with control or Snail lentiviruses were cultured for 7 days and then subjected to SA‐β‐gal staining; photographs were taken (upper panel) to count the number of SA‐β‐gal‐positive cells (lower panel). *P < 0.05, ANOVA test. (F) Panc‐1 cells treated with 1 ng·mL^−1^ of TGF‐β for 7 days were subjected to SA‐β‐gal staining; photographs were taken (upper panel) to count the number of SA‐β‐gal‐positive cells (lower panel). Each value in (C–F) represents the mean ± SD of triplicate determinations from a representative experiment. Similar results were obtained at least three independent experiments. *P < 0.05, ANOVA test.

We already reported that Snail is dramatically induced by TGF‐β in pancreatic cancer Panc‐1 cells, which harbor an activating K‐Ras mutation (G12D) 3. When Ras was silenced by specific siRNAs, Snail induction and subsequent invasion induced by TGF‐β were almost completely suppressed 3. To further determine the roles of Snail in Panc‐1 cells, we designed several siRNAs targeting Snail and transfected them into the cells. Snail siRNA#1 drastically suppressed Snail expression, Snail siRNA#2 suppressed it moderately (Fig. 1A), and the seven other siRNAs had little effect (data not shown). Upregulation of Snail by TGF‐β was also inhibited by transfection with the Snail siRNAs (Fig. 1B). Cell morphology was slightly changed, from a cobblestone‐like shape to the ‘sunny‐side up’ shape (data not shown). Because the ‘sunny‐side up’ shape is one of the characteristics of cellular senescence, we stained the cells for SA‐β‐gal following transfection with Snail siRNAs (Fig. 1C). The percentage of SA‐β‐gal‐positive cells was higher in Snail‐knockdown Panc‐1 cells than in control cells and nontransfected cells (Fig. 1C). These findings were also confirmed in pancreatic cancer MIAPaCa‐2 and Suit‐2 cells and lung cancer A549 cells (Figs 1D and S1A). By contrast, overexpression of Snail in Panc‐1 cells by lentivirus vectors reduced cellular senescence (Fig. 1E). In addition, treatment with TGF‐β increased Snail expression and suppressed cellular senescence (Fig. 1B,F), suggesting that Snail regulates cellular senescence in cancer cells.

### Snail knockdown and cellular senescence in normal fibroblasts

**Figure 2:**
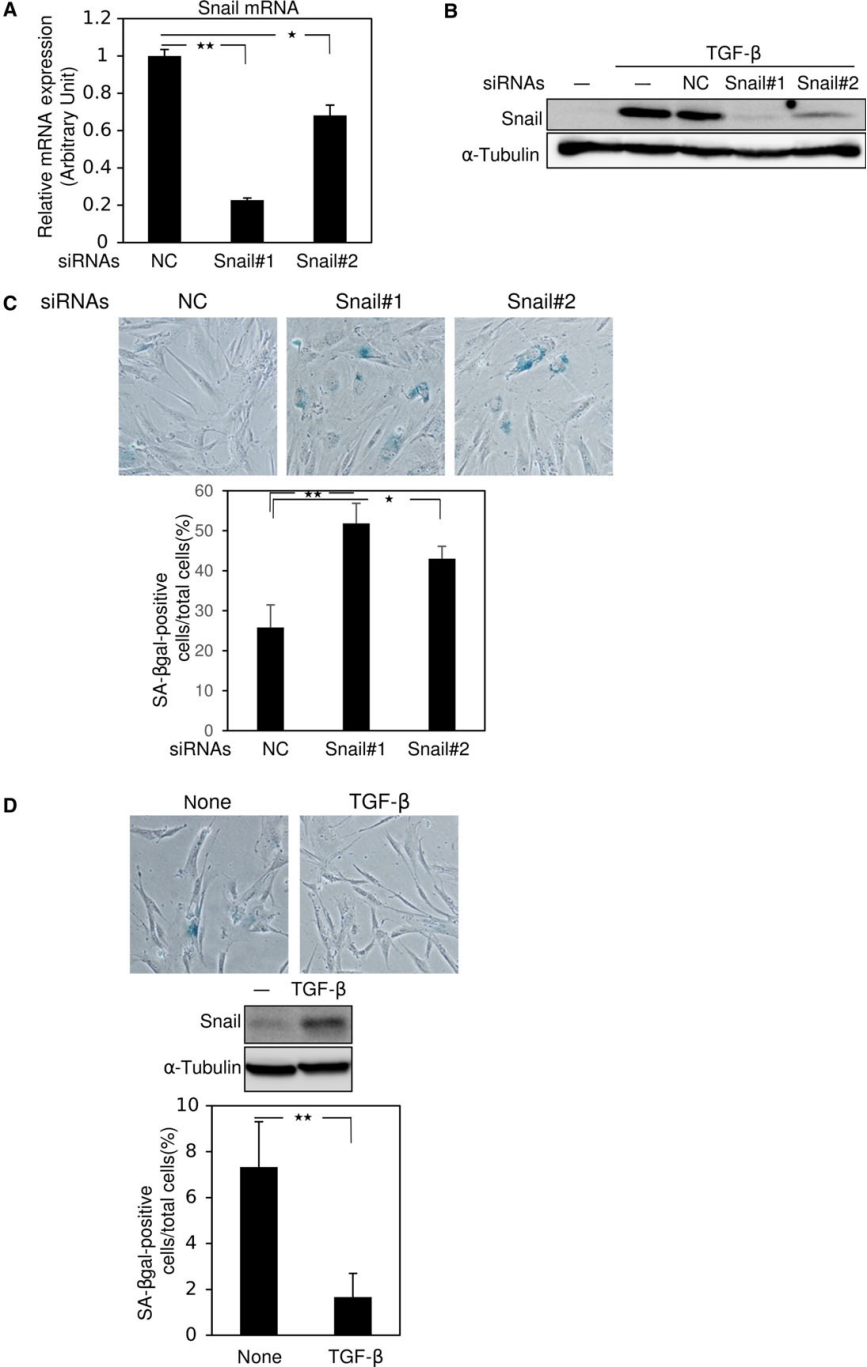
Snail knockdown induces cellular senescence in IMR90. (A and B) Thirty‐six hours after transfection of control siRNA (NC) or two kinds of Snail siRNAs (#1 and #2) into IMR90 cells, expression levels of Snail were examined by qRT‐PCR (A) and immunoblotting following treatment with 1 ng·mL^−1^ of TGF‐β for 3 h (B). α‐Tubulin levels were monitored as a loading control (B). (C) Five days after transfection with control siRNA (NC) or Snail siRNAs (#1 and #2), IMR90 cells were subjected to SA‐β‐gal staining; photographs were taken (upper panel) to count the number of SA‐β‐gal‐positive cells (lower panel). (D) IMR90 cells treated with 1 ng·mL^−1^ of TGF‐β for 7 days were subjected to SA‐β‐gal staining; photographs were taken (top panel), immunoblotting was performed (middle panel), and SA‐β‐gal‐positive cells were counted (bottom panel). Each value in (A, C, and D) represents the mean ± SD of triplicate determinations from a representative experiment. Similar results were obtained at least three independent experiments (A–D). *P < 0.05, **P < 0.01, ANOVA test.

Cellular senescence experiments are frequently performed in IMR90, a normal human fibroblast strain 24. We transfected IMR90 cells with Snail siRNAs and evaluated the induction of cellular senescence. qRT‐PCR analysis revealed a significant reduction in Snail mRNA by specific siRNAs (Fig. 2A). In IMR90 cells, treatment with TGF‐β for 3 h increased Snail protein, and upregulation of Snail by TGF‐β was also diminished by Snail siRNAs (Fig. 2B). Snail siRNAs induced cellular senescence in IMR90 cells, as determined by SA‐β‐gal staining (Fig. 2C), but treatment with TGF‐β reduced the level of cellular senescence (Fig. 2D). These findings suggest that Snail regulates cellular senescence in fibroblasts, as well as in several cancer cell lines.

### Signaling pathway involved in cellular senescence induced by Snail siRNA

**Figure 3:**
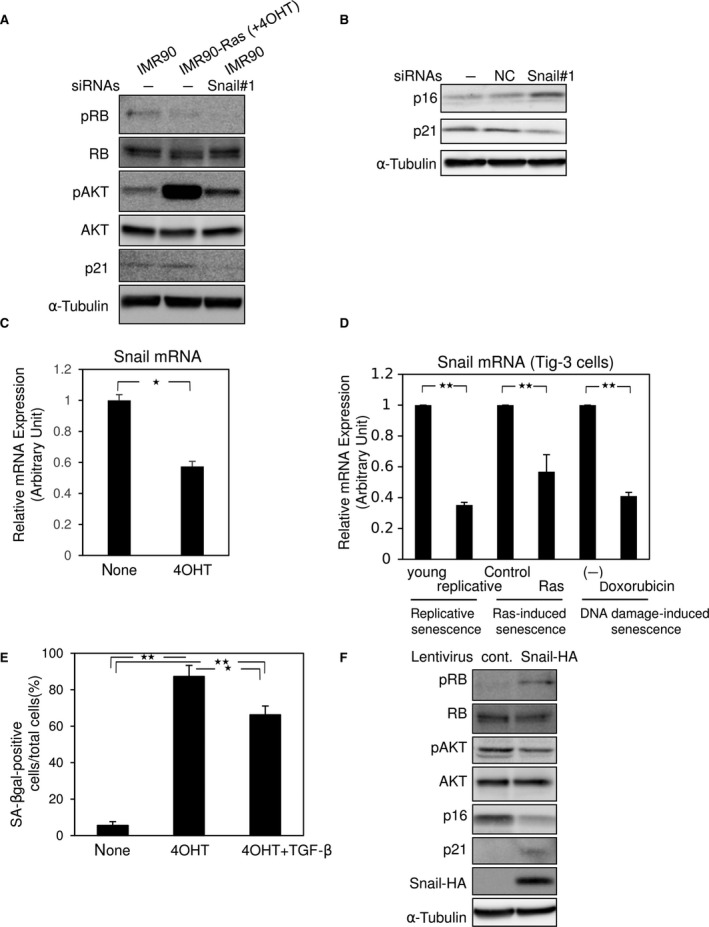
Tamoxifen induces cellular senescence in IMR90‐Ras cells. (A and B) IMR90‐Ras cells were treated with 500 nm of tamoxifen (4OHT), and IMR90 cells were transfected with control siRNA (NC) or Snail siRNA (#1). Seven days later, the cells were subjected to immunoblotting using the indicated antibodies (A and B). α‐Tubulin levels were monitored as a loading control. Results are representative of at least three experiments. (C) IMR90‐Ras cells treated with 500 nm of tamoxifen (4OHT) for 7 days were subjected to qRT‐PCR. *P < 0.05, ANOVA test. (D) Replicatively senescent, Ras‐induced senescent, and doxorubicin‐induced (DNA damage‐induced) senescent TIG‐3 cells were subjected to qRT‐PCR analyses. Young, young cells (<30 passage), replicative senescence (>70 population doubling). Young cells were treated with 250 ng·mL^−1^ doxorubicin for 10 days. **P < 0.01, ANOVA test. (E) IMR90‐Ras cells treated with 1 ng·mL^−1^ of TGF‐β and 500 nm of tamoxifen (4OHT) for 7 days were stained for SA‐β‐gal, and the positive cells were counted. Each value in (C–E) represents the mean ± SD of triplicate determinations from a representative experiment. Similar results were obtained at least three independent experiments. *P < 0.05, **P < 0.01, ANOVA test. (F) IMR90 cells infected with control (cont.) or HA‐tagged Snail (Snail‐HA) were cultured for 7 days and then were subjected to immunoblotting using the indicated antibodies. α‐Tubulin levels were monitored as a loading control. Results are representative of at least three experiments.

To investigate the mechanisms underlying cellular senescence induced by Snail siRNA, we used IMR90‐Ras cells, which express exogenous ER‐Ras protein in which Ha‐Ras (G12V) is N‐terminally fused to a 4OHT‐responsive mutant ER ligand‐binding domain 25. When the cells were treated with tamoxifen (4OHT), ER‐Ras is activated and transduces signals of the MEK–ERK pathway, leading to cellular senescence 26. As previously reported 26, IMR90‐Ras cells treated with 4OHT exhibited reduced levels of RB phosphorylation and elevated levels of AKT phosphorylation (Fig. 3A). Importantly, Snail siRNA also decreased RB phosphorylation to levels similar to those in IMR90‐Ras cells treated with 4OHT, and increased AKT phosphorylation (Fig. 3A). Consistent with previous findings that p16INK4A is upregulated during cellular senescence 17, Snail siRNA increased p16INK4A expression in IMR90 cells (Fig. 3B). Interestingly, repression of Snail mRNA was observed in IMR90‐Ras cells upon treatment with 4OHT (Fig. 3C), indicating that Ras‐induced cellular senescence in IMR90‐Ras cells partially involves Snail reduction. Downregulation of Snail was also detected in other types of senescence, namely replicative senescence, Ras‐induced senescence, and DNA damage‐induced senescence in normal human diploid fibroblast TIG‐3 cells (Fig. 3D). Similar to the findings in Figure 2D, TGF‐β inhibited Ras‐induced cellular senescence in IMR90‐Ras (Fig. 3E). Similarly, overexpression of Snail in IMR90 cells increased the levels of RB phosphorylation and decreased the levels of AKT phosphorylation, as well as p16INK4A expression (Fig. 3F). As previously reported 21, Snail overexpression and Snail knockdown upregulated and downregulated p21 expression, respectively (Fig. 3A,B,F). These findings suggest that Snail regulates cellular senescence through the AKT/p16INK4A/RB pathway, similar to cellular senescence in IMR90‐Ras cells.

### Inhibition of cell motility by Snail siRNA in cancer cells and fibroblasts

**Figure 4:**
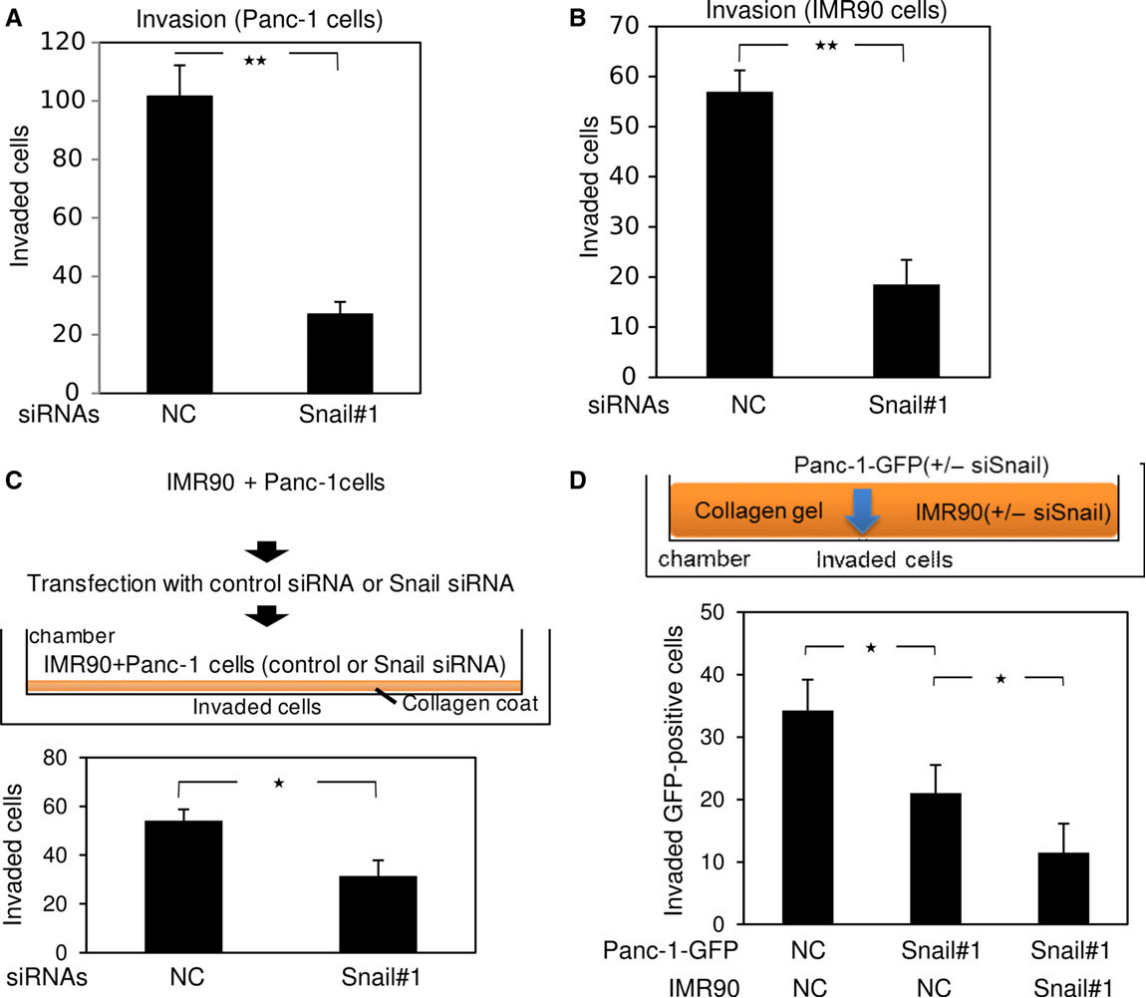
Cell motility suppressed by Snail knockdown. (A and B) Five days after transfection with control siRNA (NC) or Snail siRNA (#1), Panc‐1 cells (A) and IMR90 cells (B) were subjected to Boyden chamber assays. (C) Panc‐1 and IMR90 cells were mixed on a plate, cultured for 1 day, and transfected with control siRNA or Snail siRNA (#1). One day later, the mixed cells were seeded onto collagen‐coated Boyden chambers, and the cells that invaded the lower surfaces of the filters were counted. (D) IMR90 cells transfected with control siRNA (NC) or Snail siRNA (#1) were mixed with collagen gel and added to a Boyden chamber (shown in orange in upper panel). After Panc‐1 cells preinfected with GFP (Panc‐1‐GFP) were transfected with control siRNA (NC) or Snail siRNA (#1), the cells were seeded onto collagen gels containing IMR90 cells. Ten days later, Panc‐1‐GFP cells that had invaded the lower surfaces of the filters were counted. Results are representative of three experiments. Each value represents the mean ± SD of triplicate determinations from a representative experiment. Similar results were obtained from three independent experiments using cells transfected with control siRNA or Snail siRNA (A–D). *P < 0.05, **P < 0.01, ANOVA test.

Snail knockdown induced cellular senescence in some cancer cells, as well as in fibroblasts. In addition, Snail siRNA inhibited motility of Panc‐1, MIAPaCa‐2, and IMR90 cells without significantly affecting gelatinase expression (Figs 4A,B and S1B, and data not shown). Recent findings indicate that cancer progression is facilitated by the cancer microenvironment, in which fibroblasts or cancer‐associated fibroblasts adjacent to tumor tissue promote proliferation or invasion of cancer cells, so‐called fibroblast‐led cancer cell invasion 11, 27. Therefore, we sought to investigate the motile properties of cancer cells after transfection of Snail siRNA into both cancer cells and fibroblasts under coculture conditions. We cocultured both Panc‐1 and IMR90 cells in the same plate and forward‐transfected with Snail siRNA (Fig. 4C). One day later, the mixed cells were seeded onto collagen‐coated chambers, and the cells that invaded the lower surfaces of the filters were counted (Fig. 4C). As expected, Snail siRNA silenced its target, as determined by immunoblot analyses (data not shown), and suppressed invasion of both cell types (Fig. 4C). Next, we transfected IMR90 cells with control siRNA or Snail siRNA, mixed them with collagen gels, and added them to Boyden chambers (Fig. 4D). After Panc‐1 cells preinfected with GFP (Panc‐1‐GFP) were also transfected with control siRNA or Snail siRNA, the cells were seeded onto the collagen gels containing the IMR90 cells, and the Panc‐1‐GFP cells that invaded the lower surfaces of the filters were counted. Compared to Panc‐1‐GFP cells transfected with control siRNA, transfection of Panc‐1‐GFP cells with Snail siRNA alone moderately inhibited invasion, which was further suppressed by Snail‐silenced IMR90 cells embedded in the collagen gels (Fig. 4D). Thus, Snail expressed in fibroblasts surrounding a cancer can also assist cancer cell invasion. These findings suggest that silencing of Snail in both cancer cells and neighboring fibroblasts, but not cancer cells alone, effectively inhibits invasion of cancer cells.

## Discussion

In this study, we found that Snail siRNAs, especially Snail siRNA#1, dramatically represses its target mRNA even in the presence of TGF‐β, and induces cellular senescence in normal human fibroblasts and some types of human cancer cells. At first, to rule out the off‐target effect of the siRNAs, we infected Panc‐1 cells with lentiviral vector encoding mouse Snail to generate revertant cells. Surprisingly, the overexpressed mouse Snail was also repressed by the siRNAs, even though the sequence of the siRNAs was not completely identical, but similar, to the mouse Snail gene (data not shown). Numerous studies have reported a Snail‐mediated EMT, and one study showed that Snail siRNA causes cellular senescence in prostate cancer LNCap cells 21. However, the underlying molecular mechanisms have not been fully elucidated. The AKT/p16INK4A/RB pathway is involved in Ras‐induced cellular senescence in IMR90‐Ras cells 26. In this study, we found that the AKT/p16INK4A/RB pathway is also involved in cellular senescence regulated by Snail in IMR90 cells. Because overexpression of Snail in IMR90 cells attenuated the AKT/p16INK4A/RB signaling pathway (Fig. 3E), the change in Snail expression appears to be upstream of the AKT/p16INK4A/RB signaling pathway in the context of cellular senescence However, it remains unclear how Snail regulates phosphorylation of AKT and RB and expression of p16INK4A in fibroblasts, as well as how Snail regulates cellular senescence of Panc‐1 and MIAPaCa‐2 cells, because these cells have p16INK4A mutations. Importantly, even though p16INK4A is mutated, Snail siRNAs induced cellular senescence in cancer cells and diminished their invasive properties. Moreover, we could not exclude a possible involvement of p16 phosphorylation in cellular senescence regulated by Snail 28.

The effect of Ras on induction of cellular senescence is quite paradoxical. Activation of ER‐Ras by 4OHT transduced signals of MEK–ERK pathways 25 and induced cellular senescence partially through Snail repression in IMR90‐Ras cells (Fig. 2). Conversely, Panc‐1 cells exhibited constitutive activation of MEK–ERK pathways due to their endogenous *KRAS* mutation (RasG12D) and did not frequently undergo cellular senescence under normal culture conditions. Thus, it seems that either activation of Ras alone is not sufficient to induce cellular senescence, or there is some unknown functional difference between K‐Ras and Ha‐Ras. A previous study showed that mitochondrial defects promote cellular senescence 29 and that K‐Ras, but not Ha‐Ras, is localized to mitochondria to regulate apoptosis through interaction with Bcl‐XL. Because ER‐Ras is a protein in which Ha‐Ras (G12V) is N‐terminally fused to a 4OHT‐responsive mutant ER ligand‐binding domain 25, the localization of activated ER‐Ras in IMR90‐Ras cells should be different from that of activated K‐Ras in Panc‐1 cells. Thus, similar to the antiapoptotic effect of K‐Ras, the mitochondrial localization of this protein would impair the priming of cellular senescence. Moreover, TGF‐β suppressed cellular senescence in IMR90 and Panc‐1 cells through upregulation of Snail. A recent study reported that Smad2, a mediator of TGF‐β signaling, is a critical determinant of mitochondrial dynamics 30. Rac1b, an alternatively spliced form of Rac1, which is indirectly regulated during TGF‐β‐mediated EMT 31, causes an increase in the production of reactive oxygen species by mitochondria 32. Stat3, which facilitates Ras transformation without its nuclear accumulation 33, is also detected within mitochondria and is involved in Snail induction by TGF‐β in cooperation with Ras 22. Thus, it is possible that the TGF‐β–Smad pathway, together with Stat3 and/or Rac1b, affects mitochondrial dynamics to regulate mitochondrial localization of K‐Ras.

Notwithstanding our findings in this study, TGF‐β induces cellular senescence in human corneal epithelial cells in cooperation with NF‐kB signals 34. Consistent with this, we already reported that TNF‐α signals cooperate with TGF‐β to induce the EMT in lung cancer A549 cells harboring active K‐Ras mutation, and NF‐kB inhibitor blocks the EMT 35. In this study, we also found that Snail siRNA induces cellular senescence in A549 cells (data not shown), but the detailed molecular mechanisms by which K‐Ras status controls cellular senescence remain unclear.

In cancer microenvironments containing many kinds of cells, fibroblasts and macrophages are differentiated into CAFs and tumor‐associated macrophages (TAMs), respectively, possibly in response to cytokines or growth factors secreted from cancer cells 36, 37. These microenvironments containing CAFs and TAMs are sometimes called as reactive stroma, which promote motility and proliferation of cancer cells 38. CAFs are widely studied and known to be tumor‐promoting cells. When CAFs are coinoculated with cancer cells in mice, tumor growth is dramatically enhanced 27. CAFs promote invasion of cancer cells through a molecular mechanism called fibroblast‐led collective cancer cell invasion, which was unveiled using *in vitro* experiments 8. Thus, not only cancer cells themselves, but also CAFs or fibroblasts, are potential therapeutic targets for cancer. In this study, cellular senescence by Snail knockdown in IMR90 cells suppressed invasion of Snail‐silenced Panc‐1 cells (Fig. 4D). It is not clear whether the underlying mechanism resulted from diminished motile properties of fibroblasts, induction of cellular senescence (including the SASP) in fibroblasts, or both. However, when Snail‐silenced Panc‐1 cells were cocultivated with Snail‐silenced IMR90 cells, cellular senescence in the Panc‐1 cells was slightly, but not significantly, promoted relative to that in Snail‐silenced Panc‐1 cells cultured alone (data not shown). More importantly, siRNA against Snail induced cellular senescence and inhibited invasion of cancer cells even in the presence of p16INK4A mutations. Therefore, local administration of Snail siRNA represents a promising strategy for nucleic acid therapy in patients with cancer.

## Author contributions

SF, KE, and AT performed experiments and analyzed data. KM drafted manuscript. MS conceived and designed the project and drafted manuscript.

## Supporting information


**Fig. S1.** Cellular senescence by Snail knockdown.
