# Supplementary figures and images for: Snail suppresses cellular senescence and promotes fibroblast‐led cancer cell invasion
Source: FEBS Open Bio. 2017 Sep 11;7(10):1586–97. doi: 10.1002/2211-5463.12300 (PMC5623692; doi:10.1002/2211-5463.12300)

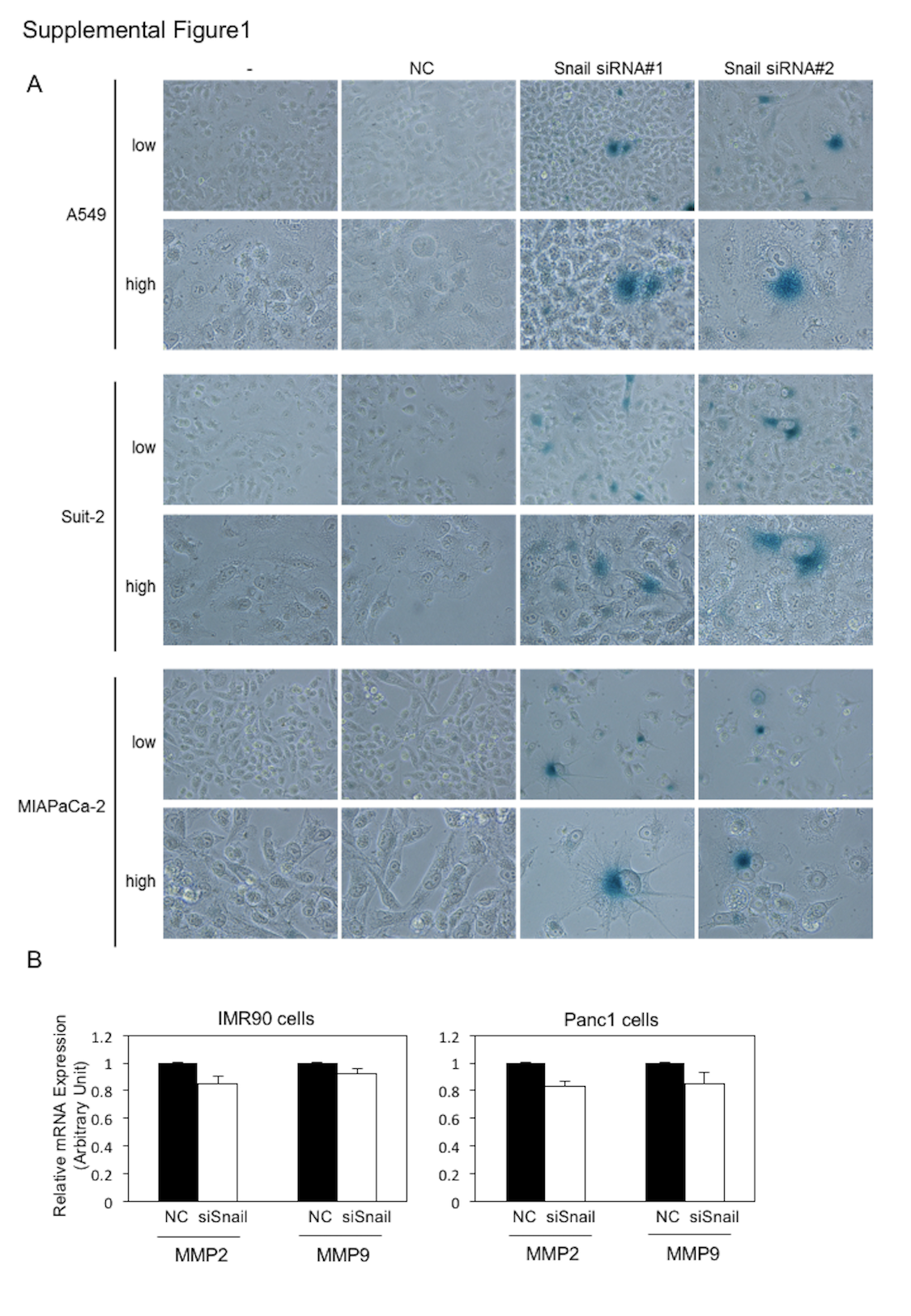

Supplement: Supplementary file 1 — Fig. S1. Cellular senescence by Snail knockdown. [file FEB4-7-1586-s001.tiff]
